# When women win, we all win—Call for a gendered global NCD agenda

**DOI:** 10.1096/fba.2021-00140

**Published:** 2022-11-25

**Authors:** Christine Ngaruiya

**Affiliations:** ^1^ Section of Global Health and International Emergency Medicine, Department of Emergency Medicine Yale School of Medicine New Haven Connecticut USA; ^2^ Yale Network for Global Noncommunicable Diseases (NGN) Yale School of Medicine New Haven Connecticut USA; ^3^ Women Lift Health Women Leaders in Global Health (2020) https://www.womenlifthealth.org/profile/christine‐ngaruiya/; ^4^ Kenyan Doctors USA https://www.kedusa.org

## Abstract

Gender is a social determinant of health, interacting with other factors such as income, education, and housing and affects health care access and health care outcomes. This paper reviews key literature and policies on health disparities and gender disparities within health. It examines noncommunicable disease (NCD) health outcomes through a gender lens and challenges existing prevailing measures of success for NCD outcomes that focus primarily on mortality. Chronic respiratory disease, one of the four leading contributors to NCD mortality, is highlighted as a case study to demonstrate the gender gap. Women have different risk factors and higher morbidity for chronic respiratory disease compared to men but morbidity is shadowed by a penultimate research focus on mortality, which results in less attention to the gap in women's NCD outcomes. This, in turn, affects how resources, programs, and interventions are implemented. It will likely slow progress in reducing overall NCD burden if we do not address risk factors in an equitable fashion. The article closes with recommendations to address these gender gaps in NCD outcomes. At the policy level, increasing representation and inclusion in global public health leadership, prioritizing NCDs among marginalized populations by global health societies and political organizations, aligning the gendered global NCD agenda with other well‐established movements will each catalyze change for gender‐based disparities in global NCDs specifically. Lastly, incorporating gender‐based indicators and targets in major NCD‐related goals and advancing gender‐based NCD research will strengthen the evidence base for women's unique NCD risks and health outcomes.

## 1 INTRODUCTION

Gender is a complex biosocial construct that constitutes a key health determinant, which interacts with other factors such as income, education, housing and affects health care access and health care outcomes. As an academic focusing on NCDs for the better part of the past decade, I have seen the effects of neglecting gender‐based disparities in this field and am alarmed. Of note, this too is colored by my own lens as a woman academic with heritage in the global South, specifically Kenya. I understand deeply and recognize the issues discussed here and in other literature on lack of representation in academia and global health leadership that continues to drive what we study or how we align our resources. I am also concerned at the lack of prioritization of gender in key indicators and outcomes, which I discuss in this paper. My greatest mantra in global health has been “what we think about, we measure.” If we are not considering gender‐based differences that affect outcomes for NCDs, we will continue to deprioritize them and consequently fail to curb the rise of and control for the burden of these diseases. The current approach will not work.

This paper reviews key literature and policies on health disparities and gender disparities within health. It examines noncommunicable disease (NCD) health outcomes through a gender lens from global studies in multiple countries. Chronic respiratory disease, one of the four leading contributors to NCD mortality, is highlighted as a case study where women have different risk factors and higher morbidity compared to men. Gender disparities in incidence, prevalence, and other measures of burden of NCDs persist yet gender dimensions remain underrepresented within the global guidelines, targets, and indicators for NCDs. The article concludes with recommended actions to achieve equitable reduction across genders in NCD morbidity and mortality. By providing some context from our past, I aim to shed light on the importance of a gendered NCD agenda for our future.

## 2 HEALTH DISPARITIES

Health disparities are defined as “differences in the incidence, prevalence, mortality, and burden of diseases and other adverse health conditions that exist among specific population groups.”^1^ The concept of health disparities dates back as early as the 1800s.^2^ Domains across which health disparities exist are well‐established in the literature, and include age, gender, race, ethnicity, level of education, employment status, level of income, and other social and economic factors.^3^, ^4^ These domains constitute independent risk factors affecting healthcare and health outcomes that are associated with worse health.^5^, ^6^ Ultimately, at the core of health disparities is an undercurrent of lack of justice.^7^ This is well encapsulated by Whitehead in her landmark paper which describes health inequities overall as “unnecessary, avoidable and unfair”.^8^

The movement to achieve equity is not new. Risk factors for health disparities have garnered attention in public health for over 30 years. In 2011, the CDC Morbidity and Mortality Weekly Report (MMWR) released the first report on health disparities, the CDC Health Disparities and Inequalities Reports (CHDIR).^9^ A 2013 publication was subsequently released, and consequently the CDC released its Strategies for Reducing Health Disparities in 2014 and 2016, respectively.^10^, ^11^, ^12^ CHDIR was the first to assess disparities across a wide range of diseases, behavioral risk factors, environmental exposures, social determinants, and health‐care access. The 2015 US Office of Minority Health Equity Summit summarized progress made since the 1985 Report of the Secretary's Task Force on Black and Minority Health (the Heckler report)^13^ and highlighted seminal events such as implementation of the Affordable Care Act, the Health and Human Services (HHS) Action Plan to Reduce Racial and Ethnic Health Disparities, and the National Partnership for Action to End Health Disparities. Additionally, in the US, disparities in healthcare have been documented in the National Health and Nutrition Examination Survey (NHANES) surveys for decades. All in all, health disparities have become commonplace in the discourse of public health practitioners, healthcare professionals, policymakers and other sectors. While many would argue that progress has been slow, and missteps continue to occur, the ability to have a shared set of language and goals to reference is a laudable first step. This is something, I would argue, is missing from the NCD agenda as it pertains to gender.

Furthermore, health disparities exist within and between countries. The report “Solid Facts” detailed the impacts of social determinants of health in the United Kingdom and Europe and served to identify opportunities for equity across government, public and private institutions, workplaces, and the community.^14^ The World Health Organization (WHO) launched the Commission on the Social Determinants of Health to assess the role of social determinants on health within and between countries in order to guide policy and other actions to address inequity.^15^ Finally, the Millennium Development Goals (MDGs) and subsequent Sustainable Development Goals (SDGs) have positioned disparities as a global priority with specific goals that include targets and indicators addressing gender equity and other disparities, which provides useful measurement tools to compare across United Nations (UN) member states.^16^, ^17^ These initiatives highlight that disparities are becoming more important to international public health organizations as well. The NCD community has an opportunity to leverage this.

## 3 GENDER DISPARITIES IN HEALTH

Gender disparities, in particular, have gained increasing recognition catalyzed by major events, such as the Beijing Declaration and Platform for Action of 1995.^18^ The declaration brought awareness to health directly and highlighted key contributors to disparities in women's health, such as education, poverty, and disparate exposure to violence. Still, action to narrow this gap continues to lag despite an unprecedented surge in visibility given to the issue.^19^

**FIGURE 1:**
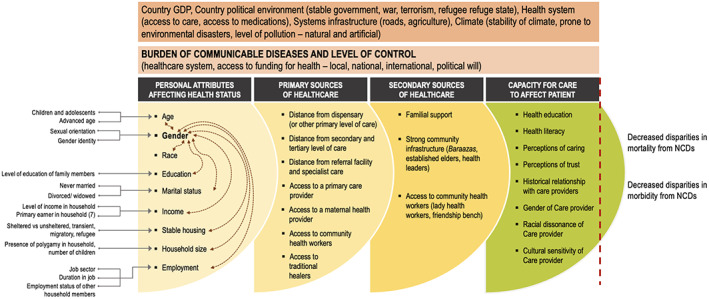
Schematic of factors associated with gender and relation to NCD mortality and morbidity.

The relationship between gender disparities in healthcare outcomes and their determinants is multi‐faceted. Figure 1 depicts some of the factors that affect NCD mortality and morbidity, and how they relate to gender, providing a schematic for thinking about and addressing this complex issue. Firstly, it demonstrates that system issues (such as sources of healthcare, and capacity for the individual to access and interact with this care) contribute additional complexities or layers to disparities. For example, if individual interventions address sensitized healthcare for transgendered patients yet fail to address distance to closest facility, then disparities in care‐seeking behaviors, quality of care, and health outcomes may still prevail. Consequently, honing in on the individual without addressing system issues may impede overall success. Secondly, it highlights the overlap or crossover, between different levels (or layers) of contributing factors affecting gender disparities. Here these factors are grouped into four larger groupings: personal attributes, primary sources of healthcare, secondary sources of healthcare, and capacity to interact with/have care affect the patient. However, they are often not as distinctly defined. This might be true for primary and secondary sources of healthcare, for example, which may be totally reversed for individuals residing in locations that are extremely remote to any healthcare facility where “secondary sources” of healthcare are in fact the only source of care. Of note, no framework will be perfect, but this does not take away from the need for a systematic way of thinking about targeting gender‐based disparities; a logic model helps unify our efforts. Thirdly, it highlights the cascading effect between individual and system levels; if one is addressed it has potentially multiplicative effects on other levels. If certain levels (or layers) go unaddressed, however, disparities may remain pervasive. For example, if interventions target the individual like off‐setting income and providing funding for healthcare, this may obviate the barrier for accessing a remote clinic/accessing healthcare. In sum, it is important to realize that as solutions are generated to address disparities at the individual level, healthcare sources and capacity for accessing them will also need to be targeted with gender differences in mind.

Fundamentally, it is also important to appreciate that gender in and of itself is a complex construct that is further delineated by associations with gender identity and sexual orientation (Figure 1, Personal Attributes). Populations outside of conventionally defined gender groups may have worse health outcomes as compared to their counterparts due to prejudice, stigma, fear of accessing healthcare, and lack of appropriate care when they do pursue it, such as in the case of transgendered populations.^20^, ^21^, ^22^ Furthermore, multiple socioeconomic factors interact with gender, including age, race, income, and employment,^23^ and therefore strategies approaching gender as a silo may fall short. Gender and race constitute a key example of the intersection of two such factors, and the importance of targeted interventions that address both concomitantly. For example, in the US, black women have higher rates of death from the two leading causes of NCD mortality, cardiovascular disease and cancer, as compared to white counterparts.^24^ In contrast, white women have a higher likelihood of dying from suicide.^25^ As a result, interventions should target the inequities affecting each respective group and their associated burden of disease, such as messaging on heart health designed specifically to most effectively reach black women and the same, respectively, for mental health affecting their white counterparts. In turn, resources from organizations should support interventions that are aligned to the respective needs of sub‐groups within a population.

The first level in Figure 1 also highlights the interaction of income, household finances, and gender, which are important to consider regarding equitable healthcare access and health outcomes. By way of example on health spending, in a secondary analysis of a national dataset in India, Maharana et al. found a marked disparity in household health expenditures on elderly individuals over age 60 when comparing men and women which denotes level of prioritization for care across the gender domain, and consequently influences outcomes. 91.2% of health expenditures went towards elderly men between 1999–2000, leaving just 8.8% of expenditures for elderly women in comparison, and consequently this changed to 85.2% of expenditures on elderly men between 2007–2008, only a slight improvement in the disparity.^26^ Disparities in food expenditure, which favored elderly men over women, was also found. This is also important given the effects of food insecurity on health outcomes such as in the case of the NCD diabetes, with a potential multiplicative negative effect on women if they have less access to food. In a different study that took place in Ghana on factors affecting access to care among males and females in one rural and one urban district, Buor found that distance and income had greater effects on health care access for females when compared to their male counterparts (see Figure 1, distance to primary source). We can conclude then that mediating barriers in access to care due to location of care facilities, distance from facilities, and leveraging cost‐reduction mechanisms should be prioritized as it pertains to women, such as through providing transportation vouchers and health insurance schemes that favor women in that context.^27^ Conversely, in the same paper, factors such as health status and quality of service affected health access among males more than females. The authors determined that the perception of their health status affected care‐seeking patterns more so for men, so they may be more likely to access care when ill. Similarly, we could then conclude that different interventions should be applied here, whereby women may benefit in this case from routine prompts to receive care, as worsened health status alone may not trigger them accessing it. Finally, in a study by Onah and Govender they also demonstrated the interaction between income and gender in Nigeria comparing Female‐Headed Households (FHH) to Male‐Headed Households (MHH). The study found that FHH, which are typically elderly, widowed, less educated, had lower household income compared to MHH and spent a higher percentage of household costs on health.^28^ Interventions that mitigate cost of care for elderly women of older age, in sum, may assist in addressing disparities in care at advanced age; such interventions have had demonstrated success in improve access of reproductive healthcare for women.^29^, ^30^

In addition, care sought by women is most often with traditional healers (see Figure 1, primary sources). Based on extensive literature on care‐seeking behaviors during pregnancy, women may have greater access to traditional medicine than to other sources of healthcare.^25^, ^31^, ^32^ This too may further impact healthcare outcomes, depending on the knowledge and services of those traditional healers. Considerations of the sources for healthcare provided across genders is important. In the end, the ability to receive and incorporate healthcare guidance will also affect outcomes (see Figure 1, capacity for care); this is a more nuanced barrier that could benefit from the use of implementation science frameworks to help identify best practices for designing and implementing interventions that optimize reach for unique groups.

## 4 GENDER GAPS IN AGGREGATE NCD OUTCOMES

At the global level, the Global Burden of Disease (GBD) study has arguably contributed the most promising data for gender disparities and other determinants of key global health priorities. NCDs constitute a significant disease burden globally affecting both mortality and morbidity according to GBD study. In the 2019 GBD study, NCDs contributed to around three out of four deaths (74.37%, 95% CI 72.82%–75.72%) and two thirds of the morbidity burden as indicated by Disability Adjusted Life Years (DALYs) (63.82%, 95% CI 61.41%–65.97%) globally.^33^ The NCDs contributing to the greatest burden of NCDs are cardiovascular disease (CVD), cancer, chronic respiratory diseases (e.g., asthma and chronic obstructive pulmonary disease), and diabetes.^34^

**FIGURE 2:**
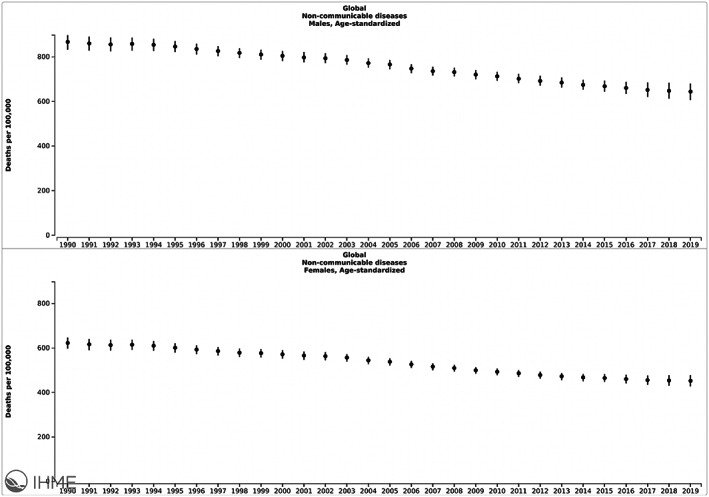
Gender disparities in global age‐standardized death rates from NCDs from 1990–2019. Image copyrighted by the Institute for Health Metrics and Evaluation. Used with permission under a Creative Commons Attribution‐NonCommercial‐NoDerivatives 4.0 International License (https://creativecommons.org/licenses/by‐nc‐nd/4.0/).

However, the disease burden is disproportionate based on gender. The GBD reveals a systematically lower death rate for women as compared to men globally (Figure 2).^35^ While the results show that the death rate due to NCDs has decreased over the past three decades for both groups, the death rate for men has persistently remained higher than that of women: 867.4 deaths per 100,000 for men vs 622.6 deaths per 100,000 for women in 1990, as compared to 644.3 deaths per 100,000 for men vs 452.2 per 100,000 for men in 2019 (Figure 2). The disproportionately higher death rate for men is likely multifactorial but could include the fact that men are less likely to seek preventative care, but instead present when disease is already advanced or when complications of disease occur, therefore resulting in higher mortality. In turn, women may be more likely to be exposed to healthcare during reproductive ages, with potential for intervention on secondary health issues including those due to NCDs. Lack of adherence to medications, when diagnosed, may also constitute another possibility for worse outcomes with NCDs among men. Also, it is important to highlight the potential for reporting bias as the access to care at the time of death, or reporting of deaths in the household, affecting datasets included in GBD analyses may favor men and result in some underreporting for women. However, I expect that this bias would likely mostly equally affect both groups; furthermore, the results originate from a large sample and with large differences still observed. These trends are also systematic, and persistent, over time so are arguably more likely to be valid.

**FIGURE 3:**
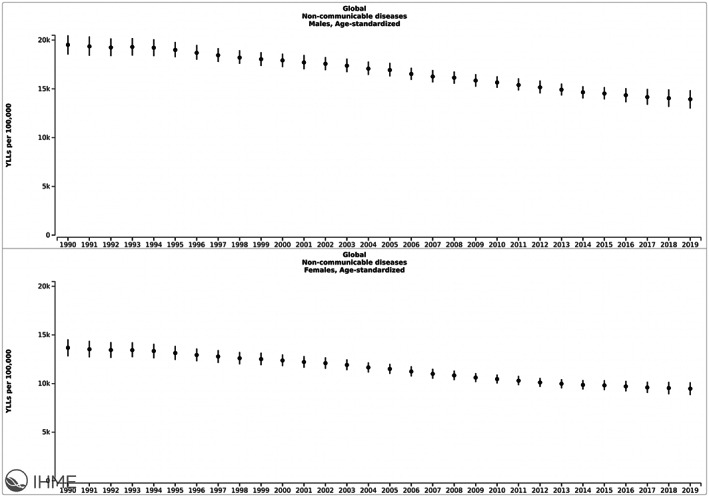
Gender disparities in global age‐standardized Years of Life Lost (YLL) from NCDs from 1990–2019. Image copyrighted by the Institute for Health Metrics and Evaluation. Used with permission under a Creative Commons Attribution‐NonCommercial‐NoDerivatives 4.0 International License (https://creativecommons.org/licenses/by‐nc‐nd/4.0/).

**FIGURE 4:**
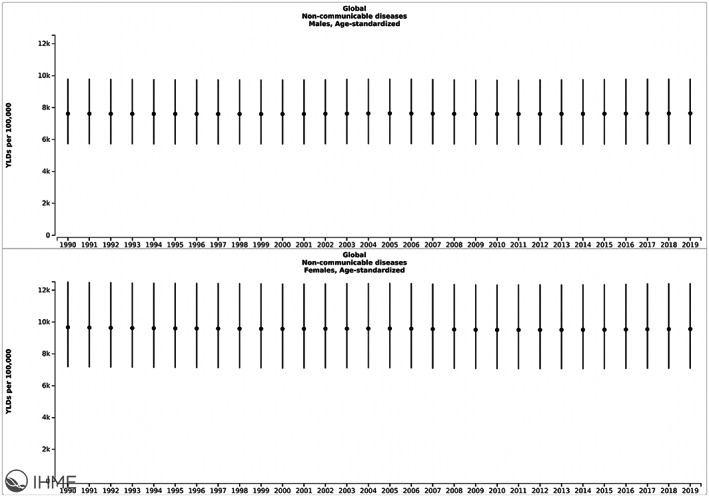
Gender disparities in global age‐standardized Years of life Lived with Disability (YLD) from NCDs from 1990–2019. Image copyrighted by the Institute for Health Metrics and Evaluation. Used with permission under a Creative Commons Attribution‐NonCommercial‐NoDerivatives 4.0 International License (https://creativecommons.org/licenses/by‐nc‐nd/4.0/).

An initial overview of GBD data at the aggregate level may be misleading as far as gender differences in NCD outcomes. While men have systematically worse morbidity as compared to women overall as measured by DALYs, the analysis reveals a more nuanced difference when morbidity is further delineated. While men tend to have higher rates of Years of Life Lost (see Figure 3), women have higher rates of Years Lived with Disability (see Figure 4). While this may be a sine qua non with women living longer than men, ultimately disease control could be optimized to reduce “disability” or morbidity resulting from the disease as defined by the GBD.^36^ Based on existing definitions, if disease control was optimized such as through appropriate treatment, disability, or perception of it would also decrease. Ultimately, this is concerning given a predominant focus by targets and indicators set out, such as those by the SDGs^37^ and the WHO NCD action plan, which focus primarily on mortality and exclude morbidity.

Additional key data and guidance on gender‐based disparities originates from *The Lancet*, which has sought to drive actionable solution through strategic organization and widespread dissemination of gender‐focused research in its journal. *The Lancet Commission on Women and Health: the key for sustainable development* ^38^ in 2015 and the recently launched *Commission on Gender and global health* ^39^ in 2020 represent a new era in academic engagement with global health and its priorities. In the original commission, the recommendations included universal healthcare for women across the lifespan, supporting and remunerating women fairly in their respective occupations, ensuring accurate representation of women in research studies, and enforcing accountability for these and other measures for gender equity.

The Lancet *Commission* in 2015 highlighted the lack of adequate attention to gender equity in diagnosing and managing NCDs among women, factors that may be impacted by lack of access to care as well as variations in classical presentations of disease as compared to male counterparts.^40^, ^41^ Solutions provided include developing sustainable health systems and building up effective primary care within these systems.^33^ The Commission highlighted the importance of the leading three risk factors, from 2013 GBD data, for mortality among women which also constitute leading risk factors for NCDs: diet, high blood pressure, and smoking.^29^, ^33^ Unmitigated risk factors can contribute to obesity and gestational diabetes in pregnancy that secondarily can lead to harmful effects on health in their progeny such as increased risk for obesity, cardiovascular disease, and diabetes later in life.^42^, ^43^

## 5 A CASE STUDY OF GENDER DISPARITIES IN CHRONIC RESPIRATORY DISEASES

Chronic respiratory disease, which is one of the leading four NCDs, encapsulates the clear gender differences in risks and outcomes discussed previously.^44^, ^45^ In 2017, the GBD found that around 550 million people (95% CI 506·9–584·9) had a chronic respiratory disease, which 40% higher than in 1990.^46^ The most common condition was chronic obstructive pulmonary disease (COPD). Men have a higher mortality rate from chronic respiratory disease as compared to women: 56.45 (54.32–58.08) deaths per 100,000 for men versus 45.97 (42.73–49.34) deaths per 100,000 for women. However, these differences disappear when comparing morbidity; the proportion of DALYs attributed to chronic respiratory disease are similar in both groups, with chronic respiratory disease constituting a 4.37% (95% CI 4.12–4.60%) proportion of all‐cause DALYs for men and 4.65% (95% CI 4.27–5.03%) proportion for women.

Furthermore, a nuanced assessment of risk of environmental exposures also indicates differences between genders. Primary risk factors associated with chronic respiratory disease include: tobacco smoke, second‐hand tobacco smoke, indoor household pollution, outdoor pollution, and occupational exposures.^47^ Men are three to five times more likely to smoke tobacco than their female counterparts.^48^, ^49^, ^50^ Furthermore, smoking tobacco is more likely to contribute to worse health outcomes in men than it is in women, as indicated by three to six times higher DALYs for men compared to women, a trend that is present regardless of the socioeconomic status of a country. Similarly, occupational exposures are higher in men.^42^

Contrary to that, women have disparate exposure to indoor household pollution with effects on higher morbidity as compared to men.^42^ So, while men are more likely to die from tobacco use and occupational exposures, women are more likely to die from indoor household exposures. These effects on women often carry over to children, as child remain in close proximity to their mothers and other women in early life.^51^ To date, more than 3 billion people still use “solid fuels” such as wood and dung, key in the role of cooking, which contribute to the continued problem with indoor household pollution.^52^ Consequently, women continue to die due to disproportionate exposures from indoor pollution due to a higher likelihood of COPD (RR 3.2, 95% CI 2.3–4.8) and lung cancer (RR 1.9, 95% CI 1.1–3.5). Recommendations addressing this are discussed later in the paper.

## 6 PROGRESS TOWARDS GLOBAL TARGETS FOR GENDER EQUITY IN HEALTH

The 2000–2015 Millennium Development Goals (MDGs) and subsequent 2015–2030 Sustainable Development Goals (SGDs) are noteworthy initiatives in setting priorities to effect change for disparities in global health. Both sets of goals account for gender. MDG 3 was to “promote gender equality and empower women” and had a target to “eliminate gender disparity in primary and secondary education, preferably by 2005, and to all levels of education no later than 2015.”^15^ Gender differences in empowerment and autonomy threaten health equity and this was reflected in MDG 3. Achievement of the targets fell short.^53^ Gender equity in education worsened during the span of the MDGs. The gap for enrollment in schools for boys and girls widened to 61 girls per 100 boys in 2011 in Sub‐Saharan Africa, which faced the greatest disparity.^54^ Additionally, disparities persist in all regions at the secondary and tertiary level. Factors contributing to disparities in education include lack of public will to support education of girls, deferential responsibility of household chores to girls, premature marriage, the cost of school fees and school supplies, and health factors such a lack of access to sanitation during menstruation and teenage pregnancy.^55^

In a meta‐analysis assessing the impacts of MDG 3 on the proposed targets of the goals and on health equity in South Asia (SA), Shannon et al found that while primary and secondary education gender gaps narrowed and parliamentary representation improved in most SA countries, there were dismal improvements in (non‐agricultural) employment with women still representing on average just 22.1% of the workforce.^56^ The health outcomes observed were conflicting because while there was a positive association between educational targets, increased employment of women, and reduced maternal mortality, they found a negative association between education and overall life expectancy as well as education and healthy life expectancy for women. It was unclear why increased education resulted in worse life and health outcomes but ultimately, the effect of the MDGs on target indicators for gender equity (including life expectancy and health) were dismal. This may also reflect the fact that other sectors may not have made concerted and iterative actions that contribute to health outcomes; education reflects just one key contributor and it cannot be addressed as a silo (see Figure 1).

The MDGs placed a focus on women in politics as an indicator of empowerment. While modest improvements in political leadership occurred during the MDG period, their evidence supported the cornerstone role of affirmative action through the use quotas as effective in ensuring appropriate representation. In fact, those countries that did not institute some form of quota in their political elections had only 12% representation of women in leadership roles, which is below the global average. However, inspired by the MDGs, UN Women, UN Development Program, and UN Population Fund developed a training program for young women politicians to be matched to senior counterparts in Uruguay, while at the same time a quota was instituted for the 2014 elections of 30% representation by women.^57^ Such capacity‐building efforts advanced equitable leadership presentation and should be further explored.

Overall, the MDG gender related targets of the MDGs were not met. First, the SDGs were ambiguous and lofty goals that were not adapted to the needs of many countries.^58^ Furthermore, many countries lacked the government capacity to adapt, implement, measure, and evaluate effective interventions. Admittedly, gender targets may also simply need more time to realize significant change and more nuanced indicators. This is reflected in some of the adaptations made in the Sustainable Development Goals. SDG 5, “Achieve gender equality and empower all women and girls,” has nine targets instead of the prior singular target, and which notably includes *policies for the promotion of gender equality and the empowerment of all women and girls at all levels* which had not been distinctly outlined previously. Given the role of policy in ensuring equity,^59^ this among the other targets, will hopefully yield dramatic improvements.

Within the SDGs, gender is addressed as inter‐sectional across multiple target indicators. SDG 5 explicitly highlights factors that interact with gender and contribute to worse health outcomes with targets acknowledging the detrimental effects of early and forced marriage, exposure to violence and lack of commensurate pay. Initiatives such as the UN Trust Fund to End Violence against women provides funding for programs addressing violence against women and girls with awards distributed to 462 initiatives in 139 countries and territories since inception in 1996.^60^ Additional funding and increased research on best practices to affect these and other determinants of equitable access, and thus healthy life expectancy for women, are needed in this vulnerable population.

In addition, it is imperative that lessons learned and best practices are shared from examples of successful or novel interventions in various countries. The MDG progress reports^61^ included some of these, but this has been more systematic for SDGs. Publications that delineate project implementation and evaluation are now centrally placed on the SDG website to help guide implementers in their roles of designing effective interventions to meet the SDG targets.^62^ Still, further infrastructure would be beneficial to making the SDGs more relevant and attainable, such as opportunity to seek out counsel, and to ensure knowledge exchange, in regular and accessible formats to improve outcomes globally such as a loose curriculum on key topics and frequent workshops. Countries could also facilitate such initiatives leveraging government, research stakeholders and academia to deliver educational programming, workshops, and discussion. Examples of this could be country‐by‐country online repositories of case studies with potential for exchange with vetted contacts. In addition, inter‐disciplinary and multi‐stakeholder seminars and workshops could be facilitated to allow for increased knowledge sharing by country, also allowing for context‐specific challenges to be addressed. This is particularly true for those countries that had significant deficiencies in achieving indicator targets.

In sum, while the MDGs and subsequent SDGs provide an unprecedented and laudable focus on health and other associated issues to advance global development, there remain opportunities to further the gender agenda under this umbrella. Clear targets that highlight disparities in gender, as well as capacity‐building specific to achieving gender equity for current health targets should be pursued. Lastly, countries must effectively bring together multisectoral stakeholders to recognize women's social determinants of health and develop gender‐sensitive interventions to advance health for all.

## 7 RECOMMENDATIONS TO REDUCE GENDER DISPARITIES IN NCDS

### 7.1 Integrate gender‐specific indicators into SDG target 3.4

SDG (Goal 3)^63^ target 3.4 seeks to “reduce by one third premature mortality from non‐communicable diseases through prevention and treatment and promote mental health and well‐being.” The indicators should be updated based on established evidence on gender disparities in the most common diseases. While men are pre‐disposed to worse outcomes from certain conditions, women are disproportionately affected in others. Women are exposed to unique diseases pertaining to pregnancy, childbirth, and menopause.^64^ They are also affected by breast cancer and cervical cancer, which have the second and eight highest cancer incidence globally of all cancers, respectively, and which constitute 10.3% of all cancer‐related deaths.^65^ Some opportunities for indicators might have included equitable screening for hypertension for both men and women,^66^ for achieving a certain prevalence of screened women for cervical cancer,^67^ and for decreased exposure of women to indoor household air pollution.^68^, ^69^, ^70^ In sum, the SDGs and other targets would benefit from gender‐specific indicators in addition to the existing overall measures; this will contribute to ensuring policy‐makers and relevant actors are both intentional but also allocate resources that reflect the mandates of these gendered guidelines. As mentioned earlier in the paper, having unified messaging or even a shared framework to align with would be ideal.

### 7.2 Foster gender equity and inclusion in the healthcare workforce

Recruiting and retaining gender‐balanced healthcare and health research workforces are key for countries; incentives to ensure this is the case should be considered. The 2015 Lancet Commission highlighted the plight of women as healthcare providers. Despite constituting the majority of healthcare providers, women tend to be underrecognized, underpaid, and their role of informal caregiving at home is underappreciated.^71^ Context‐specific considerations on barriers to care that magnify gender disparities are key in policy development and program implementation.

One needs to consider establishing equity among research scientists. The COVID‐19 pandemic elicited the strains of domestic work, childcare and other roles that traditionally burden women scientists over their male counterparts. The effects of this were clear in the reduced key authorship and overall productivity of women publishing in academic literature.^72^, ^73^, ^74^ If women scientists are not equitably represented in research, global health and discourse on gender‐based disparities in global health will not achieve full potential.

Finally, there is well‐established literature that acknowledge the role of patient‐provider relationships in ensuring target health outcomes are being realized. Multiple studies have demonstrated the role that racial/ethnic and gender concordance play in patient‐provider relationships (also see Figure 1, capacity for care), including patient perceptions of communication, level of trust, and likelihood of treatment adherence.^27^, ^75^, ^76^

### 7.3 Increase action among academic and political bodies

Medical societies have taken on gender equity and their associated issues in many specialties. The Consortium of Universities for Global Health launched a working group in 2018 called “Gender Equality in the Academic Global Community” with the intention of advancing gender equality in the workplace for women in academia.^77^ The American Medical Association has organized multiple seminars, workshops and continues to advocate for policies surrounding gender equity in health, and also is taking on gender parity in pay for women physicians.^78^ The organization also houses the John F. Giambalvo award for the advancement of women, a research grant that supports research on the advance of women physicians and trainees.^79^ The American Heart Association Go Red for Women initiative is a landmark in multiple educational, advocacy and other efforts to increase recognition of and visibility on gender equity in heart health.^80^ Pan African Women's Association of Surgeons (PAWAS) is an example of a continental group seeking to advance capacity for women surgeons and women's healthcare in Africa through networking and peer support.^81^, ^82^ Countless other medical organizations have had similar initiatives in recent years.^83^, ^84^, ^85^, ^86^ These organizations not only advocate for the status of women within their societies, but also for increased focus on gender equity for the target populations that they address. These initiatives have undoubtedly catalyzed change at the grassroots level including driving local policy change, but more is needed at institutional and national levels.^87^, ^88^

Other organizations such as Women in Global Health (WGH) founded in 2015 have sought to challenge institutional status quo through mobilizing “emerging women health leaders… to transform their own institutions.” In addition to raising awareness through events, they also providing trainings, conduct landscape analyses and have several recommendation‐based reports.^89^ Their work culminated in the signing of an MOU with the World Health Organization (WHO) for a renewed focus on gender equity including women's economic empowerment, addressing gender equity in the Universal Health Coverage agenda, and tackling both in the health workforce.^90^ Finally, WomenLift Health (WLH) seeks to bring together women and allies from across academia, policy, community, and patient levels with participants from all levels of leadership engaged. Among other programs, WLH has hosted a conference out of three international hubs: London, Rwanda, and India, and launched a trans‐national leadership capacity‐building program for women leaders. The potential for these and other coordinated international efforts to help push the needle for gender equity in global public health, including for that of NCDs, is promising.

Finally, the Lancet Commission provides a model for other academic journals to emulate in proposing, supporting, collaborating with key partners, and amplifying critical issues of health. As a primary source for exchange of ideas and catalyst for creativity in academia, academic journals have an opportunity to affect progress on global NCDs and how marginalized populations are addressed. Special issues, regular calls for papers, continued editorials and commentaries, and even commission‐style consensus driving initiatives such as the Lancet Commission efforts are some ways to continue to push the mission forward. Additionally, ensuring adequate representation of authors in addition to the topic area is key; editorial boards in global health journals will ideally prioritize the NCD issue and how it is affecting marginalized populations like women.

### 7.4 Increase data on women and the quality of that data on women

What we measure becomes the focus of implementation. Conversely, if we continue to neglect sex‐ and, in turn, gender‐based research, there will continue to be pervasive disparities in healthcare outcomes. There is fortunately an encouraging trend in the amount of research on disparities in Latin America.^91^ A growing body of work, particularly around sex‐ and gender‐based differences in cardiovascular disease patients is accumulating.^92^, ^93^, ^94^, ^95^, ^96^, ^97^, ^98^, ^99^, ^100^ While this work has brought increased visibility on the data and best practices to advance care, there is global lag in translation of findings into clinical policies and national policy. Outside of maternal child health, women's health and, importantly, gender‐based differences in health and health outcomes continue to lag.^101^ In addition, it is key to address the impacts of health system infrastructure on gender‐based differences in care and subsequent health outcomes.^102^ A need for greater attention on actionable targets that focus on reducing morbidity in addition to reducing mortality are necessary in equitably addressing the burden of global NCDs for both genders. There is an unmet need for local and national surveillance and registries that account for gender and gender differences in health outcomes for the leading burdens of disease, including NCDs.

Gender must be factored into research design and included as a distinct variable for analysis. Current limitations in the literature include lack of prioritization of gender identification in studies, inadequate power calculations to ensure representative samples across genders, and lack of consideration for equitable recruitment and retention approaches during study design. Given that women are less likely to receive NCD diagnoses,^103^ they may be less likely to be accounted for in clinical research on NCDs, or research studies being conducted at clinical sites. As such, researchers should be mindful in recruitment strategies including those that use community‐based approaches^104^, ^105^, ^106^, ^107^, ^108^ and consider the use of household‐based surveys.^109^ Sampling techniques and retention practices should also be inclusive.

Implementation science provides frameworks for inclusive research design, including gender.^110^, ^111^ However, there are often barriers in access of women for public health surveillance systems or as research study participants.^451^ These issues have been addressed head on by organizations like the US National Institutes of Health (NIH) mandate for the consideration of the inclusion of women (and minorities) in clinical trials in 1994.^112^ Such guidelines at the helm of research bodies, institutions, and organizations are laudable in driving recruitment and representation of women in datasets. Kouvari et al in 2020 provide a summary of additional practices and policies that have been implemented over the years in various nations, as well as at the UN and WHO levels that will hopefully help to mobilize this even further.^61^ In sum, policymakers and governing bodies have highlighted the issue in recent years, however, to ensure action, accountability would likely be beneficial. This may manifest as the selection of grant funding based on NCD gender or sex‐based research, more funding mechanisms that are dedicated to gender or sex‐based research overall and institutional trainings and capacity‐building for researchers on the importance of and best practices for this. Additionally, research on the effects of such policies on gender disparities for NCDs, and in health overall, could provide greater insight on how effective such policies are and the best means by which to implement and potentially enforce them.

A normalized definition of gender is needed to ensure quality and comparison of gender data. For example, the work of Pelletier et al, demonstrates that there are effects of gender on disparities in cardiovascular disease, independent of sex. Their team has done the innovative work of developing a measure for “gender”, which was developed as part of a study analyzing data from the GENESIS‐PRAXY^113^ (GENdEr and Sex determInantS of cardiovascular disease: from bench to beyond‐Premature Acute Coronary SYndrome). They identified 17 variables that were gender‐related and included these in a logistic regression using biological sex as the dependent variable. Seven variables were found to be independently associated with biological sex and included in the gender‐related score (Box 1). Scoring criteria like this and others are needed to provide more nuanced indicators for gender differences to inform more effective interventions.

BOX 1 **Components of “Gender‐related score” (Pelletier et al 2016)**

**Table:** 

Status of household primary earner;
2 Personal income;
3 Number of hours per week spent doing housework;
4 Status of primary person responsible for doing housework;
5 Level of stress at home;
6 Bem Sex Role Inventory masculinity score; and
7 Bem Sex Role Inventory femininity score

### 7.5 Amplify the voices of change for female empowerment

Gender‐based disparities have had increased focus given spotlights from initiatives such as the “me too.” movement, an advocacy initiative with roots in targeting disparate harassment and sexual violence against women, that went viral via the #metoo hashtag in 2017.^114^ “Times Up”, and its subsidiary Times Up Healthcare, were founded in 2018 after the #metoo movement call to action. As part of their efforts, they have raised money for advocacy, to support victims, and to curb harassment in various sectors of society. In turn, the traction provided by these organizations have given gender rights and gender disparities an unprecedented platform. One of the most tangible effects of these initiatives has the been the increase in women empowered to speak out against unfair treatment and their allies, with a palpable increase in the quantity, quality, tenor, and allies of efforts. The role of amplifying women's voices in effecting change for global NCDs and global health at large needs to be enacted by leveraging both political will and public support: these changes will be realized by ensuring women have platforms in the policy‐making setting, in management positions, and in grassroots advocacy and even community‐based leadership.

Women continue to be underrepresented in political leadership positions, making up only 24% of parliamentarians in a 2019 report by UN Women.^115^ This trend has seen increasing upheaval such as the majority female parliament in Rwanda,^116^ the first election of a woman president in Ethiopia's history which occurred in 2018,^117^ among other countries edging towards closing the gender gap in political leadership positions.^118^ Unfortunately, these examples are still oddities rather than the norm. All the same, they are positive harbingers and importantly so given the positive associations that have been drawn between women in leadership and health outcomes of a country. The role of women in national leadership positions has been attributed to improvement in a country's health status as well as the overall status of women in society with implications for social determinants that affect health.^119^, ^120^ In sum, women have a commitment for a “drive for equity” that is well supported in the literature. It is paramount that women lead equitably in positions of political power and other roles in which they have decision‐making capacity.

At the community level, the role of women as driver of healthcare outcomes is paramount. To that end, women need to be at the forefront of community‐based initiatives such as designing targeted interventions through community based participatory research interventions,^121^ being recognized, supported and adequately remunerated as community health workers providing education, linkage to care, and screening, and provided with the opportunity for upward mobility in leadership and decision‐making through these various roles.^122^ It is paramount that women are allowed a platform in communities, and that their stories are used to help drive change. Further enhancing women's voices in the media and other public spaces enables discourses to occur that may not otherwise be heard, and that contribute to driving culture change.^123^

### 7.6 Design program interventions to address women's environmental risks for chronic respiratory diseases

Addressing systematic differences by gender for chronic respiratory disease will require increased attention to gender differences on pathophysiology, presentation, and will require developing tailored policies and interventions to address them. Greater understanding is needed on the magnitude of the effects of varying exposures to disease, and their interactions with gender. We also need to understand the differences by which different genders access health information and which are most effective in each group.^124^ This will include understanding delivery, content, and reinforcements to mitigate harmful behaviors. For example, since women are more likely to access care from traditional healers, targeted partnerships are needed for NCD prevention and care with traditional healers We also need to determine which types of interventions are perceived as appropriate and understand factors that increase adherence to reduce exposures. While national policies need to address reducing tobacco smoke and occupational regulations, they also need to rigorously address indoor household pollution. Without addressing both sides of the spectrum, it will be impossible to achieve equitable and pervasive reduction of disease for both sub‐groups, and thus overall. Empowerment of women also plays a role given less autonomy and resources to minimize exposure to risk factors, such as indoor cooking fuel or exposure to second‐hand smoke. Therefore, opportunities that facilitate women on this topic through education and innovative measures are also necessary. Finally, as tobacco companies continue to target women and girls for marketing,^29^, ^43^ strategies need to be pre‐emptive in guarding against the rise of tobacco use using effective strategies such as those promoted by the FCTC (marketing campaigns, banning advertising), as well as for the youth such as through games or innovative school‐based programs.^125^

### 7.7 Align gender‐based NCD initiatives with other existing disparity‐focused agendas: collaboration over proliferation

Finally, in moving forward the NCD agenda overall, it has been noted by many that we are not where we hoped to be post the milestone 2011 UN High‐Level meeting – that NCDs have still been left behind.^121^ To that end, I propose that to achieve gender equity in NCD outcomes, those of us in the NCD space should further band together, and also align our mission with other longstanding initiatives such as those mentioned in the background section of this paper. Novel NCD related organizations or initiatives seemingly sprout every year; however, more could be done to coordinate these somewhat disparate, and often duplicative, efforts such as through unified consensus statements with open calls, updated research agendas, conferences and symposiums for idea exchange and to foster increased collaboration and less duplication of efforts, among other initiatives to help align and amplify the mission. Furthermore, aligning with existing, longstanding, programs, and organizations would likely bode well. For example, organizations like the CDC with new country and continental offices in various international settings outside of the US, has had a track record for decades of agenda‐setting for equitable public health. Aligning the global NCD issue as a disparities issue could be powerful. Other specialized organizations have long recognized gender‐based disparities, like the American Heart Association has for cardiovascular disease. Increasing education, awareness, and involvement with organizations like this one for the global betterment of NCD outcomes would have a multiplier effect. Certain organizations and initiatives may be country‐specific, however, as we learned with the COVID‐19 pandemic, public health issues in remote parts of the world quickly affect others directly through health or through secondary impacts that are economic or even political in nature. Leveraging these existing organizations or initiatives in countries with longstanding experience in disparities, or specifically gender‐based disparities, is low‐hanging fruit such as for shared advocacy, programming, or even collaborative research initiatives.

## 8 CONCLUSION

Identifying and addressing differences in gendered populations for targeted interventions are necessary for achieving equitable improvement in NCD morbidity and mortality globally. Key approaches will consider gender intersection with other sociodemographic factors for targeted interventions, address morbidity rather than solely focusing on mortality which may obscure NCD impacts on women, increase quality data on women in NCD research, and empower women in leadership to ensure representative policymaking, agenda‐setting, and research initiatives.
